# Intermittently Scanned and Continuous Glucose Monitor Systems: A Systematic Review on Psychological Outcomes in Pediatric Patients

**DOI:** 10.3389/fped.2021.660173

**Published:** 2021-05-05

**Authors:** Roberto Franceschi, Francesca Micheli, Enza Mozzillo, Vittoria Cauvin, Alice Liguori, Massimo Soffiati, Elisa Giani

**Affiliations:** ^1^Pediatric Unit, S. Chiara Hospital, Trento, Italy; ^2^Section of Pediatrics, Department of Translational Medical Science, Regional Center of Pediatric Diabetes, Federico II University of Naples, Naples, Italy; ^3^Humanitas Clinical and Research Center, Rozzano, Italy

**Keywords:** psychological outcomes, isCGM, CGM, type 1 diabetes, child

## Abstract

**Aim:** To explore the impact of real-time continuous glucose monitoring (rtCGMs) or intermittently scanned/viewed CGM (isCGM) on psychological outcomes in children and caregivers, and to grade the level of evidence.

**Method:** Systematic review of the literature from PubMed, Embase, Cochrane Library, Web of Science, CINAHL, Nursing reference center, Up to date, Google Scholar, and PsycINFO databases. The studies selected used validated questionnaires for investigating the psychological outcomes. We applied GRADE (Grading of Recommendations Assessment, Development and Evaluation) to rank the quality of a body of evidence.

**Results:** A total of 192 studies were identified in the initial search and after the process of evaluation 25 studies were selected as appropriate to be included in this systematic review. We found in moderate quality studies that isCGM in adolescents can improve diabetes related distress, family conflicts, fear of hypoglycemia, and quality of life, while depression, anxiety, and quality of sleep have not yet been evaluated by validated questionnaires. In moderate—high quality studies, rtCGM technology does not impact on diabetes burden, diabetes specific family conflict, and depressive symptoms. The effect on fear of hypoglycemia, sleep quality, and anxiety is still debated and RCT studies powered to find significant results in psychological outcomes are lacking. RtCGM increases satisfaction and quality of life in parents and patients wearing rtCGM.

**Conclusion:** these data present an interesting point to consider when families are deciding whether or not to start CGM use, choosing between rtCGM to reach a tighter metabolic control, or isCGM which allows greater benefits on psychological outcomes.

## Introduction

The advent of real-time continuous glucose monitoring systems (rtCGMs) or intermittently scanned/viewed CGM (isCGM) is one of the major technological innovation for the treatment of Type I Diabetes (T1D). Real-time CGM allows individuals with diabetes to follow their glucose concentration simultaneously, and to obtain information on glucose trends and trajectories. Moreover, the systems can provide warnings on upcoming hypoglycemia or hyperglycemia as well as alarms for rapid glycemic excursions (1). Meta-analyses provided evidence for real-time CGM to lower hemoglobin A1c (HbA1C) levels without increasing hypoglycemic events (1).

Importantly, recent studies confirmed that the use of isCGM has a positive impact on glucose control, by limiting glucose variability, reducing hypoglycemia, and improving long-term glucose control (2).

In addition to the stand-alone rtCGM systems, the integrated combination of pump therapy with rtCGMs allows to automatically suspend insulin delivery in the case of upcoming hypoglycemia, thus reducing or avoiding nocturnal hypoglycemia (3).

Although a clear evidence that the benefits associated with the use of rtCGMs are strictly related to a near daily use (1, 4, 5), a constant rtCGM use remains problematic for many patients in the pediatric age group (6, 7). Indeed, a better glycemic control is achieved by patients who use rtCGM for the majority of time, generally considered to be 70% or more (1, 8). Nevertheless, recent data from the Type 1 Diabetes Exchange Clinic Registry still reports that only one third of T1D-affected youth regularly wears rtCGM, although there has been an increase of use from 2013 (4% of T1D youth) to 2015 (14%) and 2017 (31%) (9). Furthermore, rtCGM wearing declines significantly over-time among T1D users (10). Barriers to a regular rtCGM use in pediatrics are reported in the following Table:

**Table:** 

**Barrier**	**Description**
Physical barriers	Pain due to sensor insertion, skin reactions to sensor, adhesive and lack of skin areas for sensor placement in young children (11, 12)
Clinical barriers	Multiple alerts and alarms can lead to alarm fatigue
Education barriers	A well-experienced diabetes team has to ensure a proper training for patients and families and a continuous support in problem solving on ways to break down barriers;
Financial barriers	Lack of insurance coverage and high costs for rtCGM supplies (13)
Psychological barriers related to rtCGM	Diabetes distress/burden, diabetes-specific family conflicts, depressive symptoms, anxiety, fear of hypoglycemia, alarm fatigue, impaired sleep quality, and quality of life (QoL).

A deeper understanding of the factors related to technologies uptake and adherence remains a crucial topic of investigation. In particular, studies on psychological factors that may predict sensor success or interruption are still limited. On the contrary, identifying psychological issues related to the sensor use would support both diabetologists in tailoring the best treatment for each patient, and youth and families in setting realistic expectations. The impact of rtCGM and isCGM on psychological outcomes in children and caregivers remains controversial (6, 14, 15). This may be due to the fact that psychological measures are usually considered as secondary outcomes in trials involving CGMs (Laffel LM 2020 JAMA, Massa GG 2019, JDRF-CGM Study Group, Diabetes Care 2010), compared to the metabolic control (HbA1c, hypoglycemia, CGM glucose metrics). Moreover, different questionnaires are used to assess the outcomes in the published studies. Also, each area of investigation (depression, fear of hypoglycemia, QoL) could be explored by different validated measures, self-reported or administered by health care providers, as summarized in Table 1 (16–42).

**Table 1:** Review of psychological measures in children used in the studies, sorted by outcome.

**Construct**	**Measure**	**Self-report or proxy-report**	**Number of items**	**Score range**	**Interpretation: ↑ score indicates**
Diabetes Burden	Problem Areas in Diabetes survey-Pediatric (PAID-Peds) (16); Problem Areas in Diabetes survey-Parent Revised (PAID-PR) (17)	Youth self-report Parent self-report	20 (PAID-Peds), 18 (PAID-PR)	0–100	↑ burden
	The Diabetes Distress Scale (T1-DDS) (18)	Parent self-report	28	Average of all 28 items, each rated on a 1–6 scale	
Diabetes-Specific Family Conflict	Diabetes Family Conflict Scale (DFCS) (19)	Youth self-report Parent self-report	19	0–100	↑ diabetes-specific family conflict
Parent Involvement	Diabetes Family Responsibility Questionnaire (DFRQ) (20)	Youth self-report Parent self-report	19	0–100	↑ parent involvement
Depressive Symptoms	Center for Epidemiologic Studies Depression Scale for Children (CES-DC) (21); Center for Epidemiologic Studies Depression Scale (CES-D) (22)	Youth self-report Parent self-report	20	0–60	↑ depressive symptoms
	The Children's Depression Inventory (CDI) (23)	Youth self-report	27	0–54	
	The Depression Anxiety Stress Scale (DASS) (24)	Parent self-report	42	0–126	
	Patient Health Questionnaire depressive scale (PHQ-8) (25)	Youth self-report	8	0–24	
State Anxiety, Trait Anxiety	Spielberger State-Trait Anxiety Inventory (STAI) (26, 27)	Youth self-report Parent self-report	20 (state), 20 (trait)	20–60	↑ anxiety
	The Diabetes Worry Scale (DWS) (28)	Youth self-report Parent self-report	50	50–250	
Fear of Hypoglycemia	Hypoglycemia Fear Survey—Worry scale (HFS) (29, 30)	Youth self-report Parent self-report	15	0–100	↑ fear of hypoglycemia
	The Hypoglycemia Confidence Scale (HCS) (31)	Parent self-report	9	0–36	
Sleep quality	The Pittsburgh Sleep Quality Index (PSQI) (32)	Parent self-report	19	0–21	↑ poor sleep quality
Youth QoL	Pediatric Quality of Life Inventory (PedsQL)—Generic and Diabetes-specific (33, 34)	Youth self-report Parent proxy-report	23 (generic), 28 (diabetes)	0–100	↑ quality of life
	Social Functioning Health Survey (SF-12) (35)	Parent proxy-report	12	0–100	
	The WHO Five Well-Being Index (WHO-5) (36)	Youth self-report Parent proxy-report	5	0–25	
	The Diabetes-Specific Quality of Life Scale (DSQOLS) (37)	Parent proxy-report	64	0–320	
	The Diabetes Quality of Life Clinical Trial Questionnaire—Revised (DQLCTQ-R) (38)	Parent proxy-report	57	0–100	
	The Appraisal of Diabetes Scale (ADS) (39)	Parent proxy-report	7	7–35	
Satisfaction with the CGM system	The CGM Satisfaction Scale (CGM-SAT) (40)	Youth self-report Parent self-report	44	44–220	↑ satisfaction with CGM use
	The Glucose Monitoring Survey (GMS) (40)	Youth self-report Parent self-report	22	44–154	
	The Blood Glucose Monitoring Communication Questionnaire (BGMC) (41)	Youth self-report Parent self-report	8	8–24	
	The Diabetes Treatment Satisfaction Questionnaire status (DTSQs) (42)	Youth self-report Parent self-report	8	0–48	

*↑increased*.

## Aim

The aim of this systematic literature review is to explore the impact of rtCGM or isCGM on psychological outcomes (diabetes distress/burden, diabetes-specific family conflicts, depressive symptoms, anxiety, fear of hypoglycemia, alarm fatigue, impaired sleep quality, quality of life, and satisfaction with the CGM system) in children and caregivers and to grade the level of evidence.

## Methods

### Criteria for Study Selection

#### Types of Studies

We included RCTs, observational studies, prospective studies, cross-sectional studies, exploratory studies, mix of qualitative, and quantitative studies. We included only published studies.

#### Types of Participants

We included patients with T1D aged between 0 and 18 years and their caregivers.

#### Types of Interventions

We included the following comparisons:

Comparison 1: rtCGM on psychological outcomes (diabetes distress/burden, diabetes-specific family conflicts, depressive symptoms, anxiety, fear of hypoglycemia, alarm fatigue, impaired sleep quality and quality of life, satisfaction) vs. capillary glucose testing for glycemic assessment in children and caregivers;

Comparison 2: isCGM on psychological outcomes (diabetes distress/burden, diabetes-specific family conflicts, depressive symptoms, anxiety, fear of hypoglycemia, alarm fatigue, impaired sleep quality and quality of life, satisfaction) vs. capillary glucose testing for glycemic assessment in children and caregivers.

Comparison 3: rtCGM vs. isCGM on psychological outcomes (diabetes distress/burden, diabetes-specific family conflicts, depressive symptoms, anxiety, fear of hypoglycemia, alarm fatigue, impaired sleep quality and quality of life, satisfaction) in children and caregivers.

#### Outcomes

Psychological outcomes in children and caregivers included: diabetes distress/burden, diabetes-specific family conflicts, depressive symptoms, anxiety, fear of hypoglycemia, alarm fatigue, impaired sleep quality, quality of life, satisfaction.

A detailed description of outcomes and related measures is reported in Table 1 (16–42).

### Search Methods

We conducted a systematic search of the literature according to the PICOS model (Population, Intervention, Comparison, Results, Study design).

**Table:** 

Population	Pediatric patients (0–18 years old) with Type I diabetes and theirs caregivers
Intervention	Use of Intermittently Scanned Continuous Glucose Monitoring (isCGM) or Real-Time Continuous Glucose Monitoring (rtCGM) Systems
Comparison	Capillary blood glucose monitoring or isCGM
Results	Variations in diabetes distress/burden, diabetes-specific family conflicts, depressive symptoms, anxiety, fear of hypoglycemia, alarm fatigue, impaired sleep quality, and QoL
Study design	RCTs, observational studies, prospective studies, cross-sectional studies, exploratory studies, mix of qualitative, and quantitative studies

The study exclusion criteria were:

- patients >18 years; patients with Type II Diabetes;
- studies not meeting the established primary and secondary outcomes;
- animal research studies;
- devices: use of closed loop systems;
- reviews, conference abstracts, full texts not available.

We did not apply language restrictions.

Sources used for literature review included: PubMed, Embase, Cochrane Library, Web of Science, CINAHL, Nursing reference center, Up to date, Google Scholar, and PsycINFO.

Articles published from 1/01/2006 to 31/12/2020 were considered for the current review. Search terms, or “mesh” (MEdical Subject Headings) for this systematic review included: “*CGM AND distress,” “CGM AND sleep quality,” “CGM AND psychological variables,” “Glucose monitoring AND distress,” “Glucose monitoring AND sleep quality,” “Glucose monitoring AND psychological variables,” “Flash glucose monitoring AND distress,” “Flash glucose monitoring AND sleep quality,” “Flash glucose monitoring AND psychological variables.”*

According to the PICOS detailed above, filters for participants' age (0–18 years), and study characteristics were activated.

### Data Extraction and Management

Two review authors independently extracted data by using the forms integrated in the sources' systems.

The following characteristics were reviewed for each included study:

- reference aspects: authorship(s); published or unpublished; year of publication; year in which study was conducted; other relevant papers cited;
- study characteristics: study design; type, duration; informed consent; ethics approval;
- population characteristics: age, number of participants;
- intervention characteristics: type, duration, mode of use of rtCGM and isCGM;
- evaluation of the outcomes as reported in Table 1 (16–42).

Disagreements were solved by discussion.

### Assessment of the Certainty of the Evidence

We used the GRADE approach (Grading of Recommendations Assessment, Development and Evaluation) to rank the quality of a body of evidence (www.gradeworkinggroup.org) for the following outcomes: diabetes distress/burden, diabetes-specific family conflicts, depressive symptoms, anxiety, fear of hypoglycemia, alarm fatigue, impaired sleep quality, quality of life, and satisfaction with the rtCGM and the isCGM systems.

Two review authors independently assessed the certainty of the evidence for each of the outcomes above. In the case of risk of bias in the study design, imprecision of estimates, inconsistency across studies, indirectness of the evidence, and publication bias, we had the option of decreasing the level of certainty by one or two levels according the GRADE guidelines (43).

The GRADE approach results in an assessment of the certainty of a body of evidence and allocation to one of four grades:

**Table:** 

High	=	Further research is very unlikely to change confidence in the estimate of effect.
Moderate	=	Further research is likely to have an important impact on confidence in the estimate of effect and may change the estimate.
Low	=	Further research is very likely to have an important impact on confidence in the estimate of effect and is likely to change the estimate.
Very low	=	Any estimate of effect is very uncertain.

## Results

A total of 192 studies were identified following the literature review. After screening, we excluded 20 records as they were duplicates. When we reviewed titles and abstracts we excluded 112 records: 9 studies were published only in abstract form, 100 studies did not investigate the outcomes of interest (Table 1), 3 studies were not available in the full text form.

A total of 60 full-text manuscripts were assessed for eligibility: 27 studies were excluded as no data were available for the analysis, besides the ones reported in the abstracts; 4 studies were excluded as they reported data from the same cohort of patients; 4 studies were excluded as they resulted to be literature reviews when the full-texts were analyzed. A final number of 25 studies, 6 on isCGM, 19 on rtCGM, were included in this systematic review.

**Figure 1:**
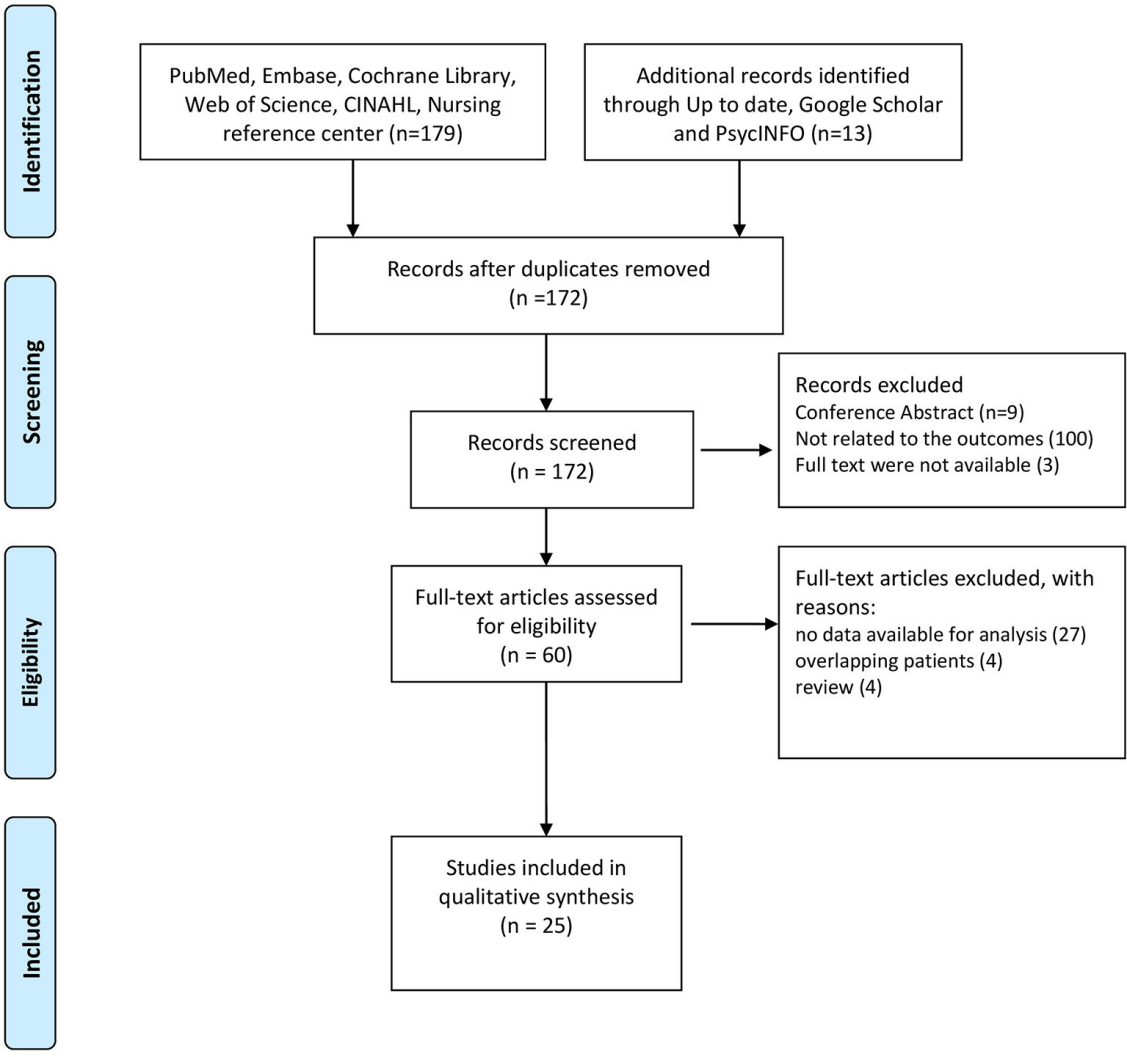
Preferred Reporting Items for Systematic Reviews (PRISMA) flow diagram showing the progress of studies through the review.

The PRISMA flow diagram in Figure 1 shows the process of study evaluation.

A summary of results from the studies included in this systematic review is reported in Tables 2, 3.

**Table 2:** Analysis of the 25 papers included in the systematic review.

**References**	**Main objective**	**Characteristics**	**Methodology**	**Main results—outcomes**	**Limits of the study and evidence level**
Al Hayek et al. (44)	Effect of isCGM on DRD	12-week prospective study 187 children and adolescents (13–19 years) with T1D, using the conventional fingerprick method. 31% were on CSII Region: Saudi Arabia	At baseline sensors were fixed. T1-DDS (diabetes distress) questionnaire was administered at T0 and + 12 weeks	T +12 weeks, in comparison to the baseline (fingerprick) showed significant decrease in all the seven the subdomains and in total T1-DDS (diabetes distress score). Increased frequency of glucose monitoring with isCGM. Substantial drop in HbA1c and in the frequency of hypoglycemia was observed.	Lack of a control group; limited number of risk factors assessed. **- Moderate -**
Boucher et al. (45)	Early experiences with isCGM	4 week qualitative study 15 participants with T1D (age 13–20 years) Device: isCGM Region: New Zeeland	Interviews 1-month from starting the isCGM. The interview analyzed: -Impacts of isCGM -Facilitators and challenges of using isCGM -Supporting patients in using isCGM	Participants perceived isCGM to be easy to use and discrete. All participants reported that isCGM alleviated burden of managing diabetes. Most (*n* = 12/15) participants perceived an improvement in their diabetes self-management. Other benefits: Facilitate to do insulin all the time Improved concentration Increased physical activity Improved sleep: reduced nocturnal hyperglycemia and helps to identify how to prevent reoccurring nocturnal hypoglycemia Less parental conflict Reduces worry about glucose level Improved social life Barriers: the most common challenges of isCGM use were: premature sensor loss, forgetting to scan, skin irritation, technical problems. All participants anticipated continuing to use isCGM	This finding may not be generalizable to longer periods of use. The sample may not be representative of the general population **- Low -**
Boucher et al. (46)	Parental perspectives after isCGM start.	Qualitative study 12 parents (age of children and adolescents with T1D: 13–20 years) Device: isCGM 11% of children used CSII Region: New Zeeland	A interview explored: -Impacts of isCGM -Facilitators/challenges of using isCGM -Supporting patients in using isCGM	The following themes were identified: (1) improved parental *well-being:* “peace of mind” while their adolescent slept; reduced diabetes-specific worry and improvement in sleep quality (2) reduced diabetes-specific parent–child conflict (3) facilitated parental role in management: easier to perform glucose checks; helped guide treatment decision isCGM has the potential to reduce diabetes management burden for both adolescents and parents. Barriers: premature sensor loss and sensor malfunction, isCGM costs.	Limitation were the small sample size. The parents included in this study were predominantly of European ethnicity and the findings may not apply to minority populations. **- Low -**
Vesco et al. (47)	Diabetes technology use on adolescent and DRD	Cross-sectional study. Adolescents with T1D (12–18 years) and parents (*N* = 1,040; primarily mothers) 64% were on CSII, 11% rtCGM+CSII Region: USA	Adolescents were categorized by technology use: rtCGM Alone, CSII Alone, rtCGM+CSII, or No Technology Adolescents (PAID-T) and parents (P-PAID-T) completed an online questionnaire	Adolescents: rtCGM use was associated with less DRD compared to No Technology, rtCGM+CSII and CSII Alone Parents: results were similar but with smaller effect size for parent-reported distress rtCGM Alone was associated with lower HbA1c compared to No Technology CSII alone and CSII+rtCGM Alone was associated with lower HbA1c compared to No Technology. rtCGM+CSII gave advantage over CSII Alone.	The sample was composed of mostly Caucasian participants from higher income families which is not representative of all youth with T1D. Small number of participants in the rtCGM Alone technology use group. **- Moderate -**
Erie et al. (48)	rtCGM practices in homes and schools, attitudes and expectations of parents and caregivers	Cross-sectional, using quantitative and qualitative methods Parents and daytime caregivers (school nurse, daycare teacher, nanny). Age of the children cared for by the respondents was 2–17 years 32 patients wore Dexcom® G4 or G5 sensors and 1 patient wore a Medtronic Enlite® Sensor Region: USA	Anonymous survey assessing characteristics of rtCGM use 57 survey pairs were distributed. 33 parents and 17 daytime caregivers responded	All parents and most caregivers (78%) reported decreased overall worry/stress. Parents felt positive about rtCGM use, it brought them peace of mind and a sense of security. Daytime caregivers felt comfortable with rtCGM and many of them felt that use of these systems allowed to work in a collaborative manner with parents to provide intensive diabetes management Frequency of sensor use was very high with 94% of respondents stating their child used the sensor 7 days a week	Relatively small sample size and response rate of 58% amongst parents and 1/3 of daytime caregivers Respondents were extremely adherent to sensor technology **- Low -**
Barnard et al. (49)	Impact of diabetes-related technology in spouses and caregivers of people with T1D	Survey, quantitative, and qualitative mix 100 parents/caregivers and 74 partners 83% of children and 72% of adults were on CSII	Participants were recruited *via* the Glu online community website. Online questions (PAID-5, WHO-5) and specific questions exploring the impact of technology	High use of rtCGM in both groups-partners and parents/caregivers. Parents/caregivers reported more negative emotions and decreased *well-being* related to their family members T1D, compared to partners, DRD was common, as was sleep disturbance associated with device alarms and fear of hypoglycemia. 87% of partners and 66% of parents/caregivers rated their own QoL as good Disrupted sleep was commonly reported with 73% of parents/caregivers and 59% of partners reporting waking because of diabetes technology. Of these, 54% of parents/caregivers and 12% of partners reported waking at least 4 times a week. The main reasons reported were rtCGM alarms and fear of hypoglycemia. False alarms were uncommon with 26 and 23%, respectively.	This study reaches only participants who are members of the Glu community (membership may be more tech savvy) as an online community **- Low -**
Kashmer et al. (50)	Characteristics of patients most willing to use rtCGM	Exploratory study Parents of children (0–18 years) with T1D responded to the online survey (no. 457) 70% used CSII Region: USA	Online survey software was utilized to administer a 50-item questionnaire to parents of children with T1D. Primary outcomes were parental interest, attitudes and concerns	Only 12% of parents whose child had previously used a rtCGM Over 90% of the parents indicated a high level of interest in having their child use a rtCGM. Primary variables related to interest in rtCGM, were use of CSII, checking BG more than six times daily and parental worry about high or low BG. Age of the child and HbA1c were not related to parental interest in a rtCGM. Only a very few parents (6%) believed that using a rtCGM would increase their diabetes-related stress. Less than 2% of parents indicated believing that they would be overwhelmed Some (7%) were concerned that they would give too much or too little insulin if they saw glucose readings continuously.	The survey instrument was not formally validated. **- Low -**
Burckhardt et al. (51)	Effect of rtCGM with remote monitoring on psychosocial outcomes in parents of children with T1D	RCT, two 3-month periods (participants spended 3 months in each of the two study arms) 49 children with T1D, 2–12 years, along with their parents	Participants “naïve” for rtCGM At the first visit and after each 3-month period, parents and children (aged 8–12 years) completed: HFS, PedsQL, DASS, STAI, PSQI, RTCGM-SAT The primary outcome was parental HFS	Parental Hypoglycemia fear scores (HFS) were lower while the child was using rtCGM with remote monitoring. Parental health-related QoL and family functioning, stress, anxiety, and sleep measures also improved significantly after intervention	Relatively small sample size **- Moderate -**
Beck et al. (14)	Impact of rtCGM on QoL among individuals with T1D	Multicenter trial RCT, 26 weeks f/up 206 children and 228 adults with T1D 110 Children on rtCGM, 106 on capillary BG. Most on CSII	HFS, PAID, SF-12 questionnaires were completed at baseline and 26 weeks by all participants and by parents (<18 years old). The rtCGM-SAT was completed by the rtCGM group (participants and parents) at 26 weeks.	Survey completion was high (rtCGM group: adults 98%, youth 93%, parents 97%; control group: 94–100%). There was substantial satisfaction with rtCGM technology after 26 weeks among participants and parents. QoL scores remained largely unchanged for both the treatment and the control group, although there was a slight difference favoring the adult rtCGM group on several subscales High baseline levels of QoL were found in this population No variation in parental burden associated with diabetes	High baseline levels of QoL in the participants who were predominantly non-Hispanic white, well-educated, privately insured, and most commonly treated with insulin pumps at enrollment **- High -**
Giani et al. (13)	Biomedical and psychosocial factors associated with rtCGM use	6 months observational study 61 T1D (8–17 years) and their parents 80% were treated with CSII Region: USA	At the first visit and after 6 months period, patients and their parents completed: HFS, DFRQ, DFCS, CES_D, STAI-CP, PAID, P-PAID, PedsQL	There was no decline in any of the psychosocial factors At baseline parents of youth using rtCGM consistently reported higher QoL for their children than the parents of youth using rtCGM less often. Youth scores were lower than parent scores for parent fear of hypoglycemia, state anxiety, traitanxiety, and diabetes burden; were higher for youth generic QoL and youth diabetes-specific QoL Youth and parent scores were significantly positively correlated for parent involvement, diabetes-specific family conflict, diabetes burden, youth generic QoL and youth diabetes-specific QoL rtCGM use declined over the 6 months	Modest sample size; the study sample presented a large proportion of participants treated with CSII and high frequency of BG monitoring at baseline, relatively low HbA1c. Therefore, the results may not be generalizable to the general population of youth with T1D. **- Moderate -**
Markowitz et al. (52)	Impact of rtCGM on psychological variables that may influence diabetes treatment adherence	RCT Children (8–17 years old) and adults, randomized to the rtCGM or BGM group for 6 months. 86% were on CSII Region: USA	49 participants were enrolled and completed at 0 and 6 months: HFS, PedsQL, SF-12, CDI, CES-D, STAI, BGM, DFCS, PAID	There were no differences in reported fear of hypoglycemia between rtCGM and BGM groups Parents in both groups reported significantly more FOH than youth. rtCGM youth and their parents and rtCGM adults reported more negative affect around BGM than the BGM group. rtCGM youth reported more trait anxiety than BGM youth, whereas rtCGM adults reported less state and trait anxiety than BGM adults. rtCGM parent-proxy report of depression was significantly higher than that reported by BGM parents. Reported levels of diabetes-specific family conflict were similar between groups.	This study was not powered to find significant result **Moderate**
Messer et al. (53)	Adolescent reported barriers to diabetes device use and to determine targets for clinician intervention	Cross-sectional study Survey on 411 adolescents (12–19 years) with T1D. 75% were on CSII Region: USA	411 adolescents completed the survey. 225 (55%) were on rtCGM Online survey with PHQ-8, PAID-Peds, SEDM, and General Technology Attitudes Survey, the Diabetes Technology Attitudes Survey	Barriers: cost/insurance related concerns; wear related issues: hassle of wearing the device, dislike of device on body Adolescents who endorsed more barriers also reported more diabetes distress, family conflict and depressive symptoms Pump and rtCGM discontinuers both endorsed more barriers and more negative perceptions of technology than current users, but reported no difference from device users in diabetes distress, family conflict, or depression.	Potential underrepresentation of adolescents not using any diabetes technology or using intermittently scanned rtCGM **- Moderate -**
Pickup et al. (54)	To analyze narratives about experiences of real-time rtCGM in people with T1D	Qualitative study 50 children with T1D (3–17 years) using rtCGM and 50 caregivers Most participants (87%) used rtCGM+CSII Region: UK	Online survey on rtCGM duration, frequency of sensor wear, funding and a free narrative about experiences or views about rtCGM. Qualitative framework analysis to analyze 100 responses was analyzed 71% used sensors ≥75% of the time	Experiences were overwhelmingly positive, with reported improved -sleep: most participants who mentioned sleep (81%) wrote that they were able to sleep more easily, with less disturbance, FOH, and a feeling of safety, with rtCGM -QoL, and physical and psychological *well-being* (reduced stress for patient and caregiver, reassurance and security, more confidence and independence, improved energy, mood, and QoL) -reduced frequency of SMBG Barriers: sensor inaccuracy and unreliability, and “alarm fatigue.” The advantages of rtCGM used with CSII with PLGM were recorded by several participants, noting reduced hypoglycemia frequency and fear of nocturnal hypoglycemia.	Responses were based on perception Participants who were funded might tend to be biased toward the positive features of rtCGM to justify the funding. **- Low -**
Telo et al. (55)	Patient and family behavioral and clinical characteristics associated with rtCGM	Cross-sectional study 358 children with T1D (age 8–18 years) Device: rtCGM 70% of patients with rtCGM used CSII, and 84% of controls Region: USA	Youth and their parents completed: DMQ, DFCS, DFRQ, PedsQL.	rtCGM group performed more frequent BGM; reported greater adherence to diabetes care; higher youth QoL; less diabetes-specific family conflict. No differences with respect to parent involvement in diabetes management. Patients who are already wearing CSII may be less reluctant	Only 28% of eligible youth who were approached for the rtCGM study agreed to wear a rtCGM device compared with 66% of the eligible general pediatric population who were approached. This probably because they recognized potential burdens related to current rtCGM technology. **- Moderate -**
Ng et al. (56)	Effects of rtCGM on patient and caregiver well-being, worry, fear of hypoglycemia and glycemic control.	12 months cohort study 16 children with T1D (age 2–17 years) Device: rtCGM (Dexcom G4®) All the patients were on pump therapy Region: United Kingdom	Children aged >12 years completed the HFS Parents completed a modified version of the HFS-P12	Improvement in fear of hypoglycemia (FOH), for both parents and children, were observed. rtCGM gave to parents and children the confidence to modify treatment regimen and rtCGM improved their anxieties, fear, and worry. rtCGM improved the children's and their parents' *well-being*. After 8 months follow up, 5 patients used rtCGM intermittently and up to 58% were not using their rtCGM routinely.	The small sample size limits transferability of the findings to the whole clinic population. **- Low -**
Burckhardt et al. (57)	rtCGM and psychosocial outcomes	2 months prospective cohort study 65 parents and 46 children with T1D (age 15 ± 1.81 years) Some patients were treated with CSII. Device: Dexcom® G5 and Medtronic Guardian Connect. Approximately 70% of the participants were using systems with remote monitoring. Region: Western Australia	To children over 12 years of age and their parents: HFS, PSQI, DTSQs, Gold Hypoglycemia awareness questionnaire after starting rtCGM	Total parental Hypoglycemia Fear and worry decreased, no difference in children were observed. Satisfaction regarding diabetes treatment improved both in parents and children Frequency of overnight BG testing decreased significantly. The percentage of children with reduced awareness of hypoglycemia decreased. Reported parental sleep quality improved Parents reported to miss fewer work days 11 children stopped rtCGM because of: sensor connection issues, general dislike, sensor falling off during exercise and problems with sensor change.	The small sample size limits transferability of the findings to the whole clinic population. Moreover, rtCGM was discontinued due to technical issues and dislike of the system. **- Moderate -**
Jaser et al. (58)	Associations between rtCGM and child sleep, glycemic control and adherence, parent sleep and well-being, parental fear of hypoglycemia, and nocturnal caregiving behavior	Descriptive observational study 515 parents of 2–12-year-old participants in the T1D Exchange clinic registry. Device: rtCGM 80% used insulin pump	Surveys were emailed to parents: CSHQ, PSQI, HFS, WHO-5 questionnaires	67% of children met criteria for poor sleep quality. Child sleep was not related to the use of diabetes-related technology (rtCGM, insulin pump) Child sleep quality and duration was related to HbA1c but not to mean frequency of BG monitoring. Children with poor sleep quality were more likely to experience severe hypoglycemia and DKA. Poorer child sleep quality was associated with poorer parental sleep quality, parental *well-being*, and fear of hypoglycemia.	Use of parent-report measures of child sleep **- Moderate -**
Al Hayek et al. (59)	Effect of isCGM on glycemic control, hypoglycemia, HTQoL, and FOH	3 months prospective study 47 youth with T1D (age 13–19 years) Device: isCGM 38% of children used CSII Region: Saudi Arabia	At the baseline and after 3 months validated questionnaires were administered: HFS-C, PedsQL 3.0 DM.	isCGM scanning can effectively reduce fear of hypoglycemia (FOH), worry and HbA1c level. It also improves QoL. The frequency of self-testing by isCGM is 8 times greater than in BGM by finger pricking. A higher frequency of isCGM scan positively correlates with behavior and QoL Significant improvement in behavior, worry, and hypoglycemia among the CSII patients.	Small sample size and inclusion of only one center for study. **- Moderate -**
Mauras et al. (6)	rtCGM benefit in young children aged 4–9 years with T1D	RCT, 26 weeks 146 children with T1D, 4–9 years 64% were on pumps Region: USA	Participants were “naïve” for rtCGM Parents completed at baseline and at 26 weeks: GMS, PAID, HFS, CGM-SAT The primary outcome was HbA1c	rtCGM wear was well-tolerated, and parental satisfaction with rtCGM was high. However, parental fear of hypoglycemia was not reduced. rtCGM wear decreased over time	**- High -**
Laffel et al. (60)	Effect of rtCGM on glycemic control and 20 secondary outcomes	RCT 153 youth with T1D (age 14–24 years), HbA1c 7.5–10.9% Device: rtCGM (Dexcom G5®) 70% of youth used CSII Region: USA	Youth completed at the baseline and after 26 weeks: PAID, HCS, PSQI	rtCGM use gave reduction in the time spent in hyperglycemia and hypoglycemia; difference in the glucose monitoring satisfaction. No difference in diabetes problem areas, hypoglycemia confidence and sleep quality were reported. The use of rtCGM device does not increased burden.	rtCGM used in the trial required twice-daily calibrations with BGM. **- High -**
Lawton et al. (61)	Participants' experiences using rtCGM.	Qualitative study 15 children aged <12; 13–15; >16 years HbA1c 7.5–10% 9 parents Device: Guardian™ Sensor 3, Medtronic 640G (100%) Region: United Kingdom	Interview, after ≥4 weeks of rtCGM use, analyzed: Previous experience of using rtCGM and SMBG; understandings, expectations and impact on diabetes self-management; likes and dislikes of the technology; views about information and training needed to support effective use of rtCGM.	Benefits deriving from the use of rtCGM: -increased awareness about glycemic values -instant and effortless access to data -prevents hypoglycemia and hyperglycemia events -short-term lifestyle changes (diet, physical activity) -better understanding of how insulin, food and physical activity impact on BG levels. -promote diabetes self-management -high treatment satisfaction Sleep quality: in some cases offered peace of mind that in target and stable BG control was being achieved and a better quality of sleep. Alarms have been identified as a factor causing decreased sleep quality and interrupted sleep. Alarm fatigue: in general individuals reported clear clinical and psychological benefits to alarms alerting. Others noted how alarms could result in distractions in the workplace or at school. Barriers: difficulty inserting and/or removing the device, finding a discreet place on the body to place it on, occasional signal loss and difficulties resulting from the need to regularly calibrate their devices (12 every hour). However, all emphasized that the clinical and psychological benefits of rtCGM outweighed any challenges encountered.	Limited observation time; CSII population, the results may not be generalizable to those using insulin injection regimens. **- Low -**
Sinisterra et al. (62)	Sleep characteristics and nocturnal BGM (NBGM) Pediatric and parental HRQOL Relationship with RTCGM use.	Prospective study, only baseline data are presented 46 parent-child dyads (age 2–5 years). Device: rtCGM Region: USA	Participants complete PedsQL Sleep quality was assessed with specific questions listed accelerometry devices were used to objectively measure child sleep for a subset of participants.	rtCGM use: -may be helpful for improving child sleep and QoL -may assist child sleep duration by minimizing their wake periods throughout the night, given that parents are less likely to wake their child up for NBGM. Parents of children on rtCGM reported a higher frequency of NBGM which may contribute to greater sleep disturbances.	This study does not include a validated parent-report sleep measure. Small sample size **- Moderate -**
Diabetes Research in Children Network (DirecNet) Study Group (63)	Psychological impact of clinical use of a rtCGM	RCT, 6 months A multi-center sample of 200 youths, aged 7–17 years, with T1D and their parents 46% were on CSII Use of the GlucoWatch G2® Biographer (GW2B) as rtCGM Region: USA	DSMP, DWS, PedsQL, CGM-SAT were administered at 0 and 6 months The DSMP was completed by telephone interview, the other on a tablet or personal computer Satisfaction with use of the GW2B was measured at end of study	Little evidence that GW2B use resulted in either beneficial or adverse psychological effects on either parents or older youths. GW2B use declined steadily during the study. Better treatment adherence (DSMP) and quality of life (PedsQL) as reported by parents at baseline was associated with more frequent GW2B use during the study.	The study was designed with the assumption that GW2B use would be relatively stable over the 6-mo study period. This was not the case as GW2B use declined steadily during the study The present study did not systematically assess how patients and parents used and responded to GW2B data **- Moderate -**
Al Hayek et al. (64)	Treatment satisfaction and sense of well-being with isCGM	12 weeks prospective cohort study 33 patients with T1D (age 14–21 years) 30% of children used CSII Device: isCGM Region: Saudi Arabia	At baseline and after 12 weeks: DTSQ and WHO-5 questionnaire	At 12 weeks: improvements in treatment satisfaction and mental *well-being* scores were detected. Improvements in the overall Diabetes treatment satisfaction questionnaire (DTSQ) score from baseline to 12 weeks. The well-being percentage score showed a statistically significant difference in *well-being* (WHO-5).	Small sample size **- Moderate -**
Pintus et al. (65)	Metabolic outcomes and QoL in children that used isCGM.	12 months prospective observational study 52 children with T1D (age 5–18 years) Device: isCGM Region: United Kingdom	The Peds QL 3.2 questionnaire was used to assess QoL before and 3 months after the use of the system. PedsQL parent report was used for parents.	The results demonstrated significant improvement in patient QoL, reduction of diabetes symptoms and treatment barriers. The use of isCGM associated with structured education improves QoL and glycemic control of children and their family.	The small sample size, limited time in observing QoL (3 months), 31–42% of patients stopped using isCGM at 6 and 12 months. **- Moderate -**

*For each study, the analyzed “Psychological Outcome” is underlined*.

**Table 3:** Summary of the evidence: rtCGM and isCGM impact on psychological outcomes in children (and parents/caregivers where specified).

**References**	**Device**	**Distress diabetes burden**	**Diabetes family conflicts**	**Depression**	**Anxiety**	**Fear/worry of hypo**	**Sleep quality**	**Alarm fatigue**	**QoL/well-being**	**Satisfaction**
Al Hayek et al. (44)	isCGM	**↓**								
Boucher et al. (45)	isCGM	**↓**	**↓**							
Boucher et al. (46)	isCGM	**↓** **↓**parents	**↓**						**↑**parents	
Vesco et al. (47)	rtCGM	**↓** **↓**parents								
Erie et al. (48)	rtCGM	**↓**caregivers								**↑**caregivers
Barnard et al. (49)	rtCGM	**↓**parents/caregivers					**↓**parents/caregivers		**↑**partners more than parents/caregivers	
Kashmer et al. (50)	rtCGM	**↓**parents								
Burckhardt et al. (51)	rtCGM	**↓**parents	**↓**		**↓**parents	**↓**parents	**↑**parents		**↑**parents	
Beck et al. (14)	rtCGM	– –caregivers				**–** **–** caregivers			**–**caregivers	**↑** **↑**parents
Giani et al. (13)	rtCGM	**–** **–** caregivers	Device: Guardian™ Sensor 3, Medtronic 640G (100%)			**–** **–** caregivers			**–**caregivers	
Markowitz et al. (52)	rtCGM	**↓** **↑**	–	**↑**adults	**↑** **↓**adults	– –adults				
Messer et al. (53)	rtCGM	**↓** **↑**	**–**	–				**↑**		
Pickup et al. (54)	rtCGM	**↓** **↑**				**↓**	**↑**parents	**↓** **↑** parents	**↑**	
Telo et al. (55)	rtCGM		**↓**						**↑**	
Ng et al. (56)	rtCGM				**↓**	**↓** **↓**parents			**↑** **↑**parents	
Burckhardt et al. (57)	rtCGM					– **↓**parents	**↑**parents			**↑** **↑**parents
Jaser et al. (58)	rtCGM					**↓**parents	–			
Al Hayek et al. (59)	isCGM					**↓**			**↑**	
Mauras et al. (6)	rtCGM					–parents				**↑**parents
Laffel et al. (60)	rtCGM	–				–	–			**↑**
Lawton et al. (61)	rtCGM						**↑** **↑**parents	**↑** **↓**		**↑**
Sinisterra et al. (62)	rtCGM						**↑** **↓**parents		**↑**	
Diabetes Research in Children Network (DirecNet) Study Group (63)	rtCGM	–			**–**			**↑**	–	–
Al Hayek et al. (64)	isCGM								**↑**	
Pintus et al. (65)	isCGM								**↑** **↑**parents	

***↓**, reduced; **↑**, increased; –, no variation; **↑↓**, heterogeneity in the response*.

## Distress/Diabetes Burden

This outcome is analyzed in 3 studies on isCGM use and in 12 studies on rtCGM use in youth and their caregivers.

In pediatric patients **isCGM** reduced psychological distress for all the domains analyzed during a 12-weeks prospective study in children/adolescents [(44), Moderate] and in a 4-weeks qualitative study in adolescents/young adults [(45), Low]. This effect was reported also in parents of children and adolescents in a qualitative study [(46), Low].

**RtCGM** reduced diabetes burden in adolescent patients according to a cross-sectional study [(47), Moderate]. A similar effect was described for caregivers in five studies [(48–50), Low, (47, 51), Moderate]. In two studies no variation in diabetes burden was found both in children and caregivers [(13, 14), High-Moderate]. Broad effects were highlighted in three studies [(52–54), Moderate-Low].

## Family Conflict in the Management of Diabetes

This outcome is measured in 2 studies on isCGM use and in 5 studies on rtCGM use in youth and their caregivers.

**IsCGM** use was associated with a reduction in diabetes specific parent-child conflict and parental conflict in patients aged 13–20 years in 2 qualitative studies [(45, 46), Low].

**RtCGM** use was associated with both a reduction in family conflicts and an improvement in rtCGMs related family functioning in 2 studies included in the review [(51, 55), Moderate]. These benefits were related to a decrease in the workload associated to blood glucose monitoring (BGM) and to an increased sense of safety [(51), Moderate]. In a RCT very similar levels of family conflict between the intervention group (rtCGM) and the control group (BGM) were found [(52), Moderate]. In other two studies no differences in family conflict were reported after the initiation of rtCGM use [(13, 53), Moderate]. The perception of a high number of obstacles and barriers related to the use of rtCGM sensors is related to a greater number of family conflicts and difficulties in managing the disease [(53), Moderate].

## Depression

Depression in youth using **rtCGM** is evaluated in two studies. In a cross-sectional study on rtCGM use in adolescents, more depressive symptoms were reported by those who faced more barriers [(53), Moderate]. In a RCT in children 8–17 years old, rtCGM parent-proxy report of depression was significantly higher than that reported by BGM parents [(52), Moderate]. Data on depression in youths using **isCGM** are lacking.

## Anxiety

This outcome is measured in 3 studies on rtCGM use in youth. In a RCT evaluating children in the age 2–12 years and their parents, parental stress level was lower in the arm using **rtCGM** compared to the control group (51, Moderate). In another study including 16 children aged 2–17 years, rtCGM use was associated with an improvement in children and parents' anxieties [(56), Low].

In a RCT study, the group of youth with rtCGM reported more trait anxiety than BGM youth, whereas rtCGM adults reported less state and trait anxiety than BGM adults [(52), Moderate].

Data on anxiety in youths using **isCGM** are lacking.

## Fear/Worry of Hypoglycemia

This outcome is measured in 1 study on isCGM use and in 14 studies on rtCGM use in youth.

Fear of hypoglycemia (FOH) was reduced by **isCGM** use in adolescents older than 12 years in a 3-month prospective study [(59), Moderate]. Similarly, **rtCGM** use reduced FOH in 16 children aged 2–12 years in a 12-month cohort study [(56), Low]. Likewise, fear associated with hypoglycemic events resulted significantly lower in parents of youth using rtCGM in several studies [(51, 57, 58), Moderate, (56), Low]. RtCGM reduced the fear of nocturnal hypoglycemia in youth when integrated with a pump that automatically suspend insulin delivery in case of hypoglycemia [(54), Low].

On the contrary, in several studies no differences were found in FOH in both youth using rtCGM/isCGM [(13, 14, 52, 57), Moderate-High] and their caregivers [(6, 13, 14, 52, 60), Moderate-High]. The fear of hypoglycemic events resulted higher in parents than in children [(52), Moderate] although the sensor use. This is probably related to the fact that not all parents have full confidence in rtCGM systems: some parents are worried that the sensor may not work properly and it does not intercept hypoglycemic events [(53), Moderate].

## Sleep Quality

This outcome is measured in 7 studies on **rtCGM** use in youth. In an observational study, overall 67% of children with T1D met the criteria for poor sleep quality; a worse child sleep quality was associated with worse metabolic control and poorer parental sleep quality. Child sleep was not related to the use of diabetes-related technology (rtCGM, insulin pump) [(58), Moderate]. About caregivers, most experimented better sleep patterns with rtCGM [(51, 54), Low-Moderate], while others reported disturbed sleep due to the presence of alarms and to the fear of hypoglycemia [(49), Low].

In a qualitative study, 9 pairs of children and parents reported improved sleep quality with the sensor use [(61), Low]. A prospective study on 46 children and their parents found that kids who used rtCGM experienced fewer sleep disturbances than those who did not, but their parents had greater sleep disturbances related to a higher frequency of nocturnal blood glucose monitoring (NBGM) [(62), Moderate]. A RCT on youth aged 14–24 years using rtCGM, reported there were no differences in sleep quality between sensors users and non-users [(60), High]. Data on sleep quality in youths using **isCGM** are lacking.

## Alarm Fatigue

This outcome is measured in 5 studies on rtCGM use in youth. Parents of children aged 3–17 years using **rtCGM** reported both positive and negative responses for alarms: helpful when signaling hypoglycemia but annoying when repeatedly sounding during the night; thus, most parents reported they would like to louder alarms [(54), Low]. In a qualitative study, most parents reported clear clinical and psychological benefits associated with alarms alerting, but others noted that alarms could interfer with daily activities in the workplace or at school [(61), Low]. While alarms could reinforce a sense of hypoglycemic safety, some individuals expressed ambivalent views, especially those who perceived alarms as signaling personal failure to achieve optimal glycemic control [(61), Low]. Two additional studies included in the review highlighted that alarms can often cause annoyance and discomfort [(53, 63), Moderate].

Day caregivers, teachers or school nurses, generally appreciate alarm systems and these are not perceived as a source of distraction or disturbance but as a tool that simplifies the management of the disease [(48), Low].

## Quality of Life/Well-Being

Four studies reported on this outcome in patients with isCGM, as well as 9 studies in patients with rtCGM. The use of **isCGM** has been reported to improve QoL in children and adolescents [(59, 64), Moderate] as well as in their parents [(46, 65), Moderate-Low].

**RtCGM** systems has been reported to improve QoL in children, for easier management of insulin dosages, diet, physical activity and in school and extra-home management [(54, 55, 62), Moderate-Low]. Similarly, in parents of youths, rtCGM has been reported to improve QoL and well-being [(51, 56), Moderate-Low].

In 3 studies included in this review no variations in QoL were found after rtCGM intervention [(13, 14, 63), Moderate-High] in youths and their parents.

Parents scores regarding the QoL are significantly higher (indicative of a less favorable QoL) than the youth's one, confirming that the perception of parents regarding the QoL of their children is less favorable than the prospects of youth regarding their QoL [(63), Moderate]. Moreover, parents/caregivers compared to partners, reported more negative emotions and decreased well-being related to their family members with T1D [(49), Low].

## Satisfaction

This outcome is measured in 7 studies on **rtCGM** use in youth. Most patients using rtCGM and their parents reported high treatment-related satisfaction [(49, 57, 61), Low-Moderate].

Three RCTs of high quality confirmed the satisfaction with rtCGM use (6, 14, 60). In the first RCT, 90% of parents of 4–9 years old children, reported a high degree of satisfaction with rtCGM: the use of rtCGM makes adjusting insulin easier, shows patterns in blood glucose not seen before, and makes them feel safer knowing that they will be warned about low blood glucose before it happens [(6), High]. In the second RCT, patients aged 14–24 years using rtCGM, reported higher glucose monitoring satisfaction compared to the BGM group over a 26-weeks study period [(60), High]. In the third RCT, in patients aged 7–17 years, satisfaction scores at 26 weeks were higher for both, youths and parents, with higher scores associated with a more frequent use of rtCGM [(14), High].

In a cross-sectional study using qualitative and quantitative methods, parents and caregivers of children aged 2–17 years, felt positive about rtCGM use [(48), Low].

Data on satisfaction in youths using **isCGM** are lacking.

## Discussion And Conclusions

A large percentage of pediatric patients with T1D experiences negative emotions, including state of anxiety, fear, discouragement, and frustration for the burden of the disease management. The use of CGM systems improves glycemic control (60) but demands for extra efforts from patients and their parents. Therefore, it is important to assess if the use of rtCGM and isCGM systems is related to psychological issues (52).

Studies on how isCGM and rtCGM impact the psychological outcomes in children and their caregivers were evaluated in this systematic review. Some limitations of the revised studies need to be addressed (Table 2):

(i) the sample size resulted small or not representative of the general population is some studies; (ii) psychological measures were included as secondary outcomes in most of the studies; thus, in some cases, the study design was not adequate to support significant results; (iii) some of the questionnaires used to measure the psychological outcomes were not previously validated. Also, questionnaires varied from one study to another.

Data on psychological outcomes in the pediatric population using isCGM systems are still limited, probably due to their recent availability on the market. The use of isCGM in adolescents can reduce psychological distress, family conflicts and fear of hypoglycemia (44, 59) and improves QoL (59, 65) as reported by a Saudi Arabia group (44) in moderate quality studies. Currently, there is no evidence of a negative impact of the isCGM system on the psychological outcomes evaluated in this review. However, results from our literature review highlighted the lack of data on depression, anxiety, and quality of sleep in pediatric patients using isCGM.

Most of the studies reported that the use of rtCGM did not increase diabetes burden in adolescents and their parents/caregivers with a moderate-high quality of evidence and using the PAID-T and P-PAID-T questionnaires (6, 13, 14, 52, 60). Likewise, rtCGM did not impact the diabetes specific family conflict, as measured by DFRQ and DFCS questionnaires in a moderate quality study (13, 52). Furthermore, rtCGM did not change depressive symptoms assessed with CDI, CES-D (13), and PHQ8 questionnaires (53).

On the other hand, rtCGM resulted improving parental anxiety in a moderate quality RCT using the STAI questionnaire by Burckhardt et al. (51). However, these results were not confirmed in a moderate quality observational study using the same questionnaire, by Giani et al. (13).

Fear of hypoglycemia remains the most common diabetes-related issue among T1D, both for youth and their parents/caregivers. In a RCT (51), parental fear of hypoglycemia (FOH) evaluated by the HFS score resulted lower in the group using rtCGM. However, other moderate-high quality studies using the HFS and HCS questionnaire did not confirm this outcome (6, 13, 14, 60).

In a RCT, adolescents' sleep quality measured with the PSQI questionnaire was not different in youth using rtCGM (60). On the contrary, parental sleep quality improved with the use of rtCGM, both when measured with the PSQI questionnaire as well by accelerometry devices in parents of adolescents and of young children, respectively (62).

Alarm fatigue was broadly evaluated in patients using rtCGM by non-validated interviews. In most cases, individuals reported clear clinical and psychological benefits to alarms setting (61), but in some contexts alarms resulted annoying and intrusive (53).

In most of the studies the perceived QoL assessed by the PedsQL in patients and caregivers, resulted improved by the use of rtCGM (55, 62). In some other studies no variations in the PedsQL were reported (13, 14), probably due to the number of variables that may influence the perceived QoL in diabetes or due to the short-term follow-up. An increased satisfaction related with the rtCGM use was assessed in both parents and youth with the DTSQ, CGM-SAT, and GMS questionnaires in moderate-high quality studies (6, 14, 51, 60).

In conclusion, the benefits of isCGM and rtCGM use on glycemic control have been previously demonstrated (1, 2, 66, 67). Findings from the studies included in this systematic review suggest that: (i) the use of isCGM in adolescents can improve diabetes related distress, family conflicts, FOH and perceived QoL; depression, anxiety, and quality of sleep have not yet been evaluated with validated questionnaires; (ii) the use of rtCGM does not impact diabetes burden, diabetes specific family conflict and depressive symptoms. The effect of rtCGM use on the fear of hypoglycemia, the sleep quality and the anxiety is still debated. Further RCT studies specifically powered to investigate psychological outcomes are needed. The use of rtCGM increases both satisfaction and perceived QoL in youth and their parents, although alarm fatigue need to be prevented with alarm targeting.

Altogether, these findings represent an interesting overview to consider when families are in the process of deciding whether or not to start CGM use.

## Data Availability Statement

The original contributions presented in the study are included in the article/supplementary material, further inquiries can be directed to the corresponding author.

## Author Contributions

RF and FM made a substantial contribution to the design of this literature review, in the acquisition of data, and their interpretation and analysis as well as in the writing of the manuscript. FM and RF selected the articles of this literary review. VC, MS, EM, and EG contributed to the critical revision of the manuscript for intellectual reasons and performed a thorough proofreading of the manuscript. All the authors have definitely approved the version to publish.

## Conflict of Interest

The authors declare that the research was conducted in the absence of any commercial or financial relationships that could be construed as a potential conflict of interest.

## Abbreviations

T1D: type 1 diabetes
DRD: diabetes related distress
FOH: fear of hypoglycemia
QoL: quality of Life
HRQoL: health related quality of life
BG: blood glucose
BGM: blood glucose monitoring
isCGM: intermittently scanned/viewed CGM
CGM: continuous glucose monitoring
FGM: flash glucose monitoring
CSII: Continuous subcutaneous insulin infusion
PLGM: predictive low glucose management.
